# Teaching of reflection in higher education: a narrative review

**DOI:** 10.12688/mep.20389.1

**Published:** 2024-07-09

**Authors:** Tharin Phenwan

**Affiliations:** 1 School of Health Sciences, University of Dundee, Dundee, Scotland, DD1 4HN, UK

**Keywords:** Reflection, Review, Undergraduate, medical students, nursing students, professionalism

## Abstract

**Background:**

Healthcare professional students (HCPs) are encouraged to utilise reflection during and after their study programmes as a part of their life-long learning skill and professional competencies. However, the way in which the concept of reflection is taught and its’ influence students’ capacity to reflect have not been fully explored. This narrative review aimed to explore how the concept of reflection is taught in higher education and how the teaching of reflection influences HCP students’ capacity to reflect.

**Methods:**

Articles that were published during 2014–2024 within three databases, PubMed, CINAHL and ERIC were searched. 1929 articles were eligible for screening. 93 articles were further assessed for eligibility.

**Results:**

18 articles were included. The included articles were geographically well-distributed in both the Global North and Global South countries, indicating universal interest in the topic. Eight articles had qualitative study designs; six had quantitative designs and four had mixed-method designs.

Conceptually, the teaching of reflection could be categorised as a spectrum, ranging from: i) structured format (reflective templates or debriefing); ii) semi-structured format (physical/virtual small group discussions, video recordings, AI generative arts, in-verse reflection and concept mapping) and iii) flexible and creative (art-based pedagogy or narratives).

All included articles indicated students actual and perceived better understanding of reflection. This claim is supported both quantitatively and qualitatively via either validated instruments or narratives and themes based on students’ textual outputs.

**Conclusions:**

This review identified several teaching methods that help facilitating students’ capacity to reflect. Findings are unable to recommend the most efficient way to teach reflection since it depends on students’ progress in their programmes. However, a more relational approach to teaching of reflection is recommended. Students might begin their reflective journey with a structured format of teaching of reflection then gradually move to less-rigid format of the teaching to empower students’ autonomy.

## Introduction

Reflection is one of the metacognition skills that enhances lifelong learning capacity as well as professional competencies ^1– 3^. As such, any practice to enhance capacity to reflect is highly encouraged, particularly in healthcare professions education since it relates to better learning outcomes and long-term professional performances ^2, 4– 7^. In the UK, reflective practice (RP) is encouraged by several professional bodies such as the General Medical Council and the Nursing and Midwifery Council (NMC) as a strategy to foster healthcare professionals (HCPs)’ competencies ^8, 9^.

Still, the concept of reflection is relatively abstract hence it is challenging to frame the appropriate way to teach the concept to students, particularly those who are new to the concept ^6, 10– 12^. Furthermore, reflection could occur in various contexts and activities such as during small group discussions, online learning environment, writing or art-based activities ^12– 15^ thus making it harder to capture.

Additionally, students in higher education - particularly in the UK - have become increasingly diverse ^16^. This includes students from non-traditional background, international students or students who disclosed as disabled hence may need additional support for their learning ^17^. Consequently, the notion of Equality, Diversity and Inclusivity (EDI) in the teaching of reflection warrants further exploration to scrutinise if the existing teaching methods sufficiently address such diversity or not.

There is a need to systematically understands the way in which the concept of reflection is taught in higher education and how it influences students’ capacity to reflect hence, a narrative review was conducted. This narrative review aimed to:

- explore how the concept of reflection is taught in higher education
- explore the teaching of reflection and its influences over HCP students’ capacity to reflect

## Methodology

A narrative review was undertaken as a part of the author’s study for UKPSF’s fellowship title. The justification was to balance between the time constraints within the module (four months) and the robustness of the searching process. Although some researchers argue that a narrative review may be prone to bias, subjectivity and is not replicable ^18^, this argument is less relevant to this review and strategies have been applied to mitigate these limitations. Given that this review aims to explore a conceptual and broader understanding of how reflection is taught in higher education as well as its influence on students, a more focused approach such as a systematic review is not appropriate; instead, a broad approach such as a narrative review is deemed more appropriate. Next, the notion of ‘bias’ is not relevant to this review since the phrase is deeply rooted with post-positivist research paradigm that considers knowledge to be quantifiable, measurable and repeatable ^19^; all of which do not relate to aims of this review. 

The search terms and strategies, developed with support from an academic librarian, are described below to ensure that the search process is transparent and replicable.

The PCC framework (Population, Concept, and Context) were used to frame the scope of the review ^20^:

***P**opulation: undergraduate healthcare professionals students*

***C**oncept: teaching of reflection or reflective practice*

***C**ontext: Higher education institutes and their associated clinical placements or hospitals, colleges, global context*

The inclusion criteria were any peer-reviewed empirical studies that were published between 2014–2024 and were published in English that discussed the influence of reflection teaching methods with students. The focus was any undergraduate HCPs students in the context of higher education settings, including any associated clinical placements.

Articles were excluded if they were reviews, grey literature or focused on any theoretical discussions, debates or opinion pieces as well as dissertations and theses. Articles that did not focus on reflection teaching methods, higher education settings, were published before 2014 or were published in non-English were excluded (see Table 1).

**Table 1.** Inclusion and exclusion criteria.

Inclusion criteria	Exclusion criteria
-Peer-reviewed empirical studies (quantitative, qualitative or mixed-method designs) that discussed the influence or effectiveness of reflection teaching methods	-Reviews -Grey literature, policies, opinion piece, debate, theoretical discussion -reflection of certain experiences which did not include the influence of effectiveness of reflection teaching methods
-Articles that focussed on undergraduate healthcare professional students	-Articles that did not focus on undergraduate HCPs students
-Higher education settings -Clinical placements associated with higher education institutes	-Any contexts beyond higher education
-Articles published in English	-Non-English Articles
-Articles published between 2014–2024	-Articles published before 2014

From December 2023 to March 2024, three databases were searched: PubMed, Cumulative Index to Nursing and Allied Health Literature (CINAHL) and Educational Resources Information Center (ERIC). Eight duplicated articles were removed (see Table 2). One article was retracted by the journal due to systematic manipulation of the publication process and was subsequently removed from the screening process. Five articles were not accessible, leaving 1929 articles for title and abstract screening. 93 articles were further assessed for eligibility with the full-text reading.

**Table 2.** Search terms.

Database	Search terms	No. of articles found
PubMed	((reflect*) AND ("medical student" or "nursing student" or "student nurse" or "healthcare education" **or "dental student")) AND ((2004/1/1:2024/2[pdat]) AND (english[Filter])) AND ((y_10[Filter]) AND ** **(english[Filter])) Filters: in the last 10 years, English**	1255
	(((reflect*) AND ("medical student" or "nursing student" or "student nurse" or "healthcare education" or "dental student")) AND (universit* or "higher education" or college*) AND ((2004/1/1:2024/2[pdat]) AND (english[Filter])) AND ((y_10[Filter]) AND (english[Filter]))) AND (teach*) Filters: in the last 10 years, English	337
	((reflect*) AND ("medical student" or "nursing student" or "student nurse" or "healthcare education" or "dental student")) AND (universit* or "higher education" or college*) AND ((2004/1/1:2024/2[pdat]) **AND (english[Filter])) Filters: in the last 10 years, ** **English**	1143
CINAHL Plus	(reflection or reflective or reflective practice) AND (medical students or nursing students or healthcare professional students) AND (university or college or higher education)	1126
	(reflection or reflective or reflective practice) AND (medical students or nursing students or healthcare professional students) AND (university or college or higher education) Limiters - Publication Year: 2014–2024; English Language Expanders - Apply equivalent subjects Search modes - Boolean/Phrase	588
ERIC	(reflection or reflective or reflective practice) AND (medical students or nursing students or healthcare professional students) AND (university or college or higher education) Limiters - Published Date: 20140101-20241231 Expanders - Apply equivalent subjects Narrow by Language: - english Search modes - Boolean/Phrase	212
	(reflection or reflective or reflective practice) AND (medical students or nursing students or healthcare professional students) AND (university or college or higher education)	393

The Inclusion and exclusion criteria were applied and 76 articles were excluded:

- 24 articles were not related to students’ capacity to reflect
- 20 articles were not related to teaching of reflection
- 16 articles were not related to undergraduate HCPs students
- 12 articles were opinion pieces, debates, dissertations and student theses

**Figure 1.**
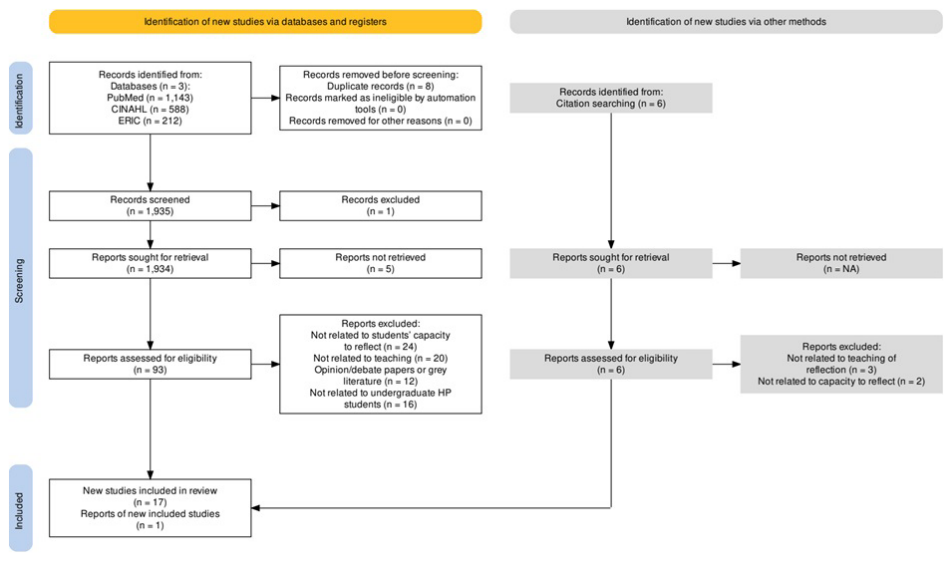
PRISMA flow diagram.

Six articles also were manually searched from the reference lists; five articles were excluded and one was included for the final analysis ^2^. Together, the final numbers of the included articles were eighteen (see Figure 1) ^21^.

## Data extraction

Data from the included articles were extracted into Microsoft Excel. The extraction process was iterative and the focus of the extracted data was revised to ensure that they were relevant to the aims of the review. The final data extraction file included:

Title, Author’s name, Year, Country, Type of study, Aims, Teaching methods employed, Data collected/generated, Data analysis techniques, Underlying theory/pedagogy, Participants, How the teaching methods influence reflection, Themes and Limitations of the study.

## Data analysis

Narratives were used to describe the findings. The author read and reread the included articles to see the patterns, similarities and differences within them. The focus related to the aims of the review. Given that there were diverse types of study from several countries within different groups of students, the underlying theory and pedagogical approach was examined to conceptually capture the findings in a more meaningful way. The included articles were not critically appraised since this review did not intend to determine the quality of each article and intended to explore a broad range of existing practices.

## Findings

### General description of the included articles

The included 18 articles were published from 2014 to 2024. There was a well-distributed geographical distribution of the included studies. The majority of the included articles (5) were conducted in the United States ^5, 22– 25^, followed by the UK (3) ^26– 28^. Two articles were conducted in Australia ^10, 29^. One article each originated from The Netherlands ^30^, New Zealand ^15^, Norway ^31^, Singapore ^32^, Spain ^33^, South Korea ^34^, Taiwan ^35^ and Thailand ^2^.

Eight articles had qualitative study designs. Six articles had quantitative designs and four had the mixed-method designs. The HCPs students in these articles were also diverse. Almost all of the articles predominantly focused on students from one field which were medical students (8), nursing students (8) and healthcare assistants (1). Only one study focused on the first-year students from various fields ^35^, indicating a universal interest of reflection from the educator’s standpoint from various disciplines.

The most frequently used underlying theory employed in these articles was Constructivism (4) followed by social constructionism (2), Sociocultural learning theory (1), Social cognitive theory (1) and Sociocritical paradigm (1). Kolb’s experiential learning theory were mentioned in two articles. Seven articles did not explicitly mention their underlying learning theory utilised; two were implied to utilise positivism research paradigm and five were implied to base on constructivism (see Extended data 1).

## Results

This section respectively discusses the findings and relates them back to the aims of this review.

### How the concept of reflection is taught in higher education

**Figure 2.**
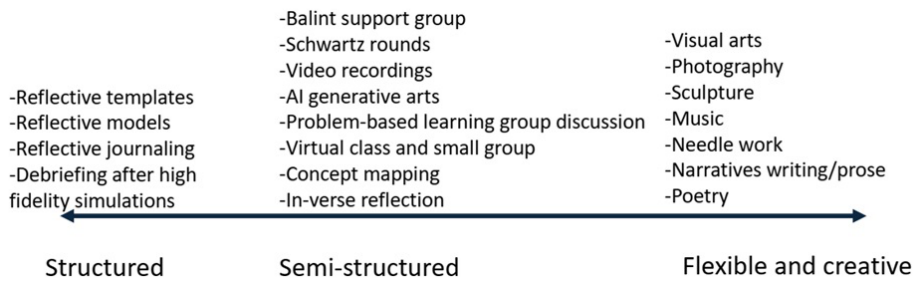
The approaches to reflection teaching methods.

Based on the included articles, the teaching of reflection could be categorised as a spectrum, ranging from:

ⅰ) structured format of reflection
ⅱ) semi-structured format of reflection
ⅲ) flexible and creative format of reflection (see Figure 2)

The structured format of reflection approach usually involves any teaching with reflective templates or reflective models that students could use to enhance and demonstrate their reflection. The reflective models used were Gibb’s reflective model ^2^ and Bain’s reflective framework ^10^. Often, structured tools such as a reflective journaling ^34^, or post-activities debrief were offered to students to facilitate on how they could express their reflection. In such instances, reflection was often not the main focus of the activities but students could demonstrate their increased capacity to reflect as well. This mainly occurred in high fidelity simulations with debriefing ^5, 10, 32^ which suggested multiple benefits of the learning activities.

This approach, whilst perceived as useful, was paradoxically seen by students as rigid. Students from several articles expressed a dislike of this approach since it was perceived as inauthentic and repetitious ^15, 22, 30^. Pedagogically, students could even become a reflective zombie ^36^; that is, they might emulate the reflective process but did not actually reflect thus making it more challenging to ascertain the authenticity of their reflection ^1^.

Alternatively, the semi-structured format of reflection provides more flexibility to students to express their thoughts but still offers scaffolding of their reflection. This approach includes Balint student support group ^28^, Schwartz round ^27^, video recording of students’ performance ^31^ and AI generative arts ^25^, problem-based learning group discussions ^22^, virtual classrooms ^23^ and concept mapping ^30^ and in-verse reflection workshops ^29^.

The teaching methods under this approach tend to include group learning experience, utilise social constructionism and offer several methods for students to express their reflections e.g., either verbally or in writing. The process almost always includes an extensive preparation for the students before during and after the sessions and is mainly dependent on facilitator’s skill to lead the sessions. For instance, Reed *et al.* (2023) piloted a novel approach to integrate the use of generative AI to facilitate nursing students’ reflection. The learning process included students, who are also the coauthors of the article, creating AI prompts to create photos that were related to nursing. They were subsequently encouraged to write and reflect on the photos and subsequently joined a small group discussion.

As the description attest, the process is rather laborious and comprised of several preparations, making this approach relatively time and resource extensive. This limitation is similar to what Gleeson *et al.* (2020) acknowledged. That is, they agreed with the usefulness of the medical Schwartz round to enhance students’ capacity to reflect yet found it financially and logistically challenging to facilitate the sessions thus may not be applicable to scale within their institution or beyond.

Finally, the flexible and creative format of reflection almost always relates to art-based pedagogy. This approach leverages the use of art forms to empower students to creatively express their reflections without any constraints. The studies included an offer to use various creative outlets namely poetry, visual arts, narrative prose, photography, sculpture, music, needlework ^15^. Or, one alternative approach over the formal reflection form of writing was offered such as poetry ^26^ or narrative writing ^24, 33, 35^. Still, despite the creativity freedom within this approach that empower student’ agency, some expressed concern over their learning process since they found it harder to express themselves or could not fully relate its relevance to a more ‘formal’ reflective assessment which tends to be dominated by a written format ^15, 26^. This was compounded from the educators’ standpoint; that is, it is impossible – and even inappropriate – to assess the quality of students’ reflection with this approach due to their subjectivity. As such, the flexible and creative format may be suitable for learning activities that do not involve any assessment.

### How the teaching of reflection influences students’ capacity to reflect

All of the included articles reported that students had either an improved understanding of reflection or improved reflective capacity. This claim was usually asserted by validated instruments to demonstrate students’ improved capacity to reflect before and after the class such as the Groningen Reflection Ability Scale (GRAS) ^22, 23, 32^, or the Reflective Thinking Level ^34^. Zhang *et al.* (2020) conducted a study to assess 63 third year nursing students GRAS post-class and one week after the class after their high-fidelity simulations with the use of video-assisted debrief. Results showed that participants had significantly improved their debriefing reflective abilities (p<0.01) after the video-assisted debrief intervention from the median of 84 to 87 pre- and post-class; this trend is also similar to other included studies, indicating the usefulness of the teaching methods employed.

For qualitative or mix-method studies, students’ textual outputs or interview transcripts were often used to support the claim that they have a better understanding of reflection. McBain *et al.* (2015) offered 14 fourth year medical students to freely choose their medium to reflect which could be either a traditional reflective essay or more creative options such as poetry, visual arts, or sculpture; their textual commentaries that accompany their works were used for the analysis. Participants universally expressed that creative outputs were more effective for them to express emotion or ideas that are difficult to articulate. This is particularly important since these students were doing a clinical rotation in a palliative care department which may be rather emotionally demanding, indicating that it might be useful to have certain tools to ease the cognitive load of participants when they were reflecting ^24, 31, 33^. Students also stated that the arts created were helpful since they could reflect in a different way that was not as repetitious or perceived as just another reflective essays ^15^. Still, most of the studies tend to capture participants’ self-perceived of enhanced reflection. Or it could come from educators’ perception that these students had a better capacity to reflect which might be challenging to verify.

### Suggestions to enhance the teaching of reflection

Findings from this review indicate that there are several approaches to enhance student’ capacity to reflect. This could be achieved either via a more structured approach which could be perceived as repetitious and inauthentic ^15, 30^. Or, students could be offered a semi-structured approach or even more creative and flexible form of reflection to empower their autonomy ^24, 26^.

Despite the various approaches to teach reflection, both the educators and students suggested that there is a need to balance between being directive and being flexible ^28, 30^. That is, for students who are relatively new to the concept e.g., those who are in the first year of their programme or have limited exposure to clinical experience, it might be more appropriate to have a structured guidance to scaffold their learning process ^29, 32^. Conversely, those who are in their later years or are more experienced reflective practitioners may find the structured reflective format repetitive or even restrictive to their reflection. Consequently, a more flexible and creative expression might be an alternative option for students to demonstrate their reflection.

### Strengths and limitations

To the author’s knowledge, this is the first review that has explored the way in which the concept of reflection is taught in higher education for HCPs students. The review demonstrates a comprehensive view of the teaching methods available that educators could consider and apply the most appropriate ones to their contexts. The review process was also thoroughly described hence making it transparent and replicable.

Still, this review poses few limitations:

First, despite the attempt to outline the screening process, this review was conducted by one person. As such, there might be certain articles that the author has overlooked. Second, the review only included articles that were published in English. This further perpetuating the dominant Western-centric scholarship, restricting the latest call for citational justice and may not be fully transferable to wider contexts ^37^. Still, this review was conducted with a limited time and resource hence the author could not include more diverse scholarly outputs that were not in English.

Third, although this review did not intend to appraise the quality of the included articles, some articles indicated either poor study designs or superficial level of analysis that lack criticality. Also, almost all of the included studies except two were conducted with a relatively small sample size from a single institute thus makes it even more challenging to transfer the findings to other contexts.

## Conclusions

This narrative review has identified several teaching methods that help facilitating HCPs students’ capacity to reflect. Even though findings are unable to recommend the most efficient way to teach reflection, it might be more appropriate to consider students’ learning needs and adjustments the teaching method accordingly. This could be achieved via a more relational approach to teaching that might begin with a structured format of reflection which gradually increases students’ autonomy to express their reflection throughout. Given that there is no one-size-fits-all to teaching due to different leaning contexts, the focus should be how educators could scaffold the students’ learning process to empower them to become a lifelong reflective practitioner.

## Data Availability

No data associated with this article. Discovery: Extended data for Teaching of reflection in higher education: a narrative review ^38^ http://doi.org/10.15132/10000254 This project contains the following extended data: Included studies Data are available under the terms of the Creative Commons Attribution 4.0 International license (CC-BY 4.0).
