# Supplementary material for: Teaching of reflection in higher education: a narrative review
Source: MedEdPublish (2016). 2024 Jul 9;14:53. [Version 1] doi: 10.12688/mep.20389.1 (PMC11316171; doi:10.12688/mep.20389.1)
Supplement: Peer review history

# Reviewer report: Reviewer response for version 1

**DOI:** 10.21956/mep.21819.r38143

**Published:** 2024-08-09

**Authors:** Tanisha Jowsey

**Affiliations:** 1 Bond University, Robina, Queensland, Australia

Hi there, thanks for undertaking this work, which I read with interest. 

**Here are recommendations to make this solid paper even better:**

 The title and introduction suggest your review is of the entire higher education field; we need clarity on the scope of your study throughout. I suggest you reword the title from higher education to 'undergraduate health professions students' and hone the focus of your introduction to match that focus.

**Abstract**

 methods suggest replacing this sentence: "Articles that were published during 2014–2024 within three databases, PubMed, CINAHL and ERIC were searched. 1929 articles were eligible for screening. 93 articles were further assessed for eligibility." I suggest this instead: "I searched three databases (PubMed, CINAHL and ERIC) for articles published between 2014–2024 using the following search terms and their derivatives: XXXX    1929 articles were eligible for title and abstract screening. 93 articles were further assessed (full text screening) for eligibility."

**Main text**

 Methods is fine. I found it unusual and insightful that you've applied a narrative focus to the included studies but I didnt see a evidence of this unique method in the way the results are presented. Did I miss something?

 Results - Ah yes, the reflective zombie - a constant frustration for teachers (!) I found Reed's approach to this - the Gen AI image reflection - a great example of one possible avenue to engage students and get them out of zombie mode. 

 The paragraph about "creative format of reflection almost always relates to art-based pedagogy" - I was surprised that creative outputs as evidence of reflection were deemed impossible to assess. There are whole schools of practice that do exactly that. For example, critical arts therapy. I myself published a book called Medicine Reflections (2017 ^1^ ) where we showcased how creative elements in the reflective portfolios of our undergrad medical students were assessed. I would suggest that it can be done. The question of whether this is appropriate or not is another matter. Perhaps you could rework those last two sentences of the paragraph to split out whether you can assess from whether you should assess?

 The remaining sections read well. Thanks.

Is the review written in accessible language?

Yes

Are all factual statements correct and adequately supported by citations?

Yes

Are the conclusions drawn appropriate in the context of the current research literature?

Yes

Is the topic of the review discussed comprehensively in the context of the current literature?

Partly

Reviewer Expertise:

I have used critical reflection in assessment in medicine for the past 12 years and currently supervise a doctoral student who is researching the role of critical reflection in paediatric surgery training. We have a published literature review in this space called 'Reflecting on insight and insights into reflection: a systematic review of insight and reflection in post graduate medical education.' I have also published a book called Medicine Reflections.

I confirm that I have read this submission and believe that I have an appropriate level of expertise to confirm that it is of an acceptable scientific standard.

## References

[1] https://researchspace.auckland.ac.nz/handle/2292/40112 , Medicine Reflections,Jowsey.T. *Compassion Publishers* .2017;

# Reviewer report: Reviewer response for version 1

**DOI:** 10.21956/mep.21819.r38149

**Published:** 2024-08-07

**Authors:** Weeratian Tawanwongsri

**Affiliations:** 1 Walailak University, Thai Buri, Nakhon Si Thammarat, Thailand

Well-written article. Your insights into this interesting topic are enlightening and comprehensively supported by citations. The use of easily understandable language makes the content accessible, and the conclusions drawn are appropriate in the context of the current research literature. The comprehensiveness and clarity of your article make it a valuable resource.

**Some suggestions:**

 1. The title may be edited to reflect the specific issue on healthcare professional students/learners.

 2. The methodology is well-written, constructed, and valid.

 3. In the discussion section, the author may provide a subsection about further considerations or gaps in studies that need to be explored.

Is the review written in accessible language?

Yes

Are all factual statements correct and adequately supported by citations?

Yes

Are the conclusions drawn appropriate in the context of the current research literature?

Yes

Is the topic of the review discussed comprehensively in the context of the current literature?

Yes

Reviewer Expertise:

None

I confirm that I have read this submission and believe that I have an appropriate level of expertise to confirm that it is of an acceptable scientific standard.
